# Dr. Charles S. Wood

**Published:** 1890-03

**Authors:** 


Dr. Charles S. Wood, of New York, died at his home, February 1, 1890. Dr. Wood was a conspicuous character in the medical circles of the period. He was a prominent surgeon in the war of the rebellion, the writer having served with him in the army of the Potomac where his best services was performed, and personally knew of his skill and value. After the war, Dr. Wood resumed civil practice in New York, was elected president of the Medico-legal Society, and subsequently served as president of several local medical societies. He was a clear thinker, a plain, straightforward, common-sense man, and a true and able physician. 'Tis sad to part with such men.

